# Determinants of moult haulout phenology and duration in southern elephant seals

**DOI:** 10.1038/s41598-021-92635-9

**Published:** 2021-06-25

**Authors:** Leandri de Kock, W. Chris Oosthuizen, Roxanne S. Beltran, Marthán N. Bester, P. J. Nico de Bruyn

**Affiliations:** 1 Department of Zoology and Entomology, Mammal Research Institute, University of Pretoria, Private Bag X20, Hatfield, 0028 South Africa; 2 Marine Apex Predator Research Unit, Institute for Coastal and Marine Research and Department of Zoology, Nelson Mandela University, Port Elizabeth, 6031 South Africa; 3 Department of Ecology and Evolutionary Biology, University of California Santa Cruz, 115 McAllister Way, Santa Cruz, CA 95060 USA

**Keywords:** Climate-change ecology, Population dynamics

## Abstract

Phenological shifts are among the most obvious biological responses to environmental change, yet documented responses for Southern Ocean marine mammals are extremely rare. Marine mammals can respond to environmental changes through phenological flexibility of their life-history events such as breeding and moulting. Southern elephant seals (*Mirounga leonina*) undergo an obligatory annual moult which involves the rapid shedding of epidermal skin and hair while seals fast ashore. We quantified the timing (phenology) and duration (the time from arrival ashore to departure) of the moult haulout of 4612 female elephant seals at Marion Island over 32 years. Using linear mixed-effects models, we investigated age, breeding state and environmental drivers of moult timing and haulout duration. We found no clear evidence for a temporal shift in moult phenology or its duration. Annual variation in moult arrival date and haulout duration was small relative to age and breeding effects, which explained more than 90% of the variance in moult arrival date and 25% in moult haulout duration. All environmental covariates we tested explained minimal variation in the data. Female elephant seals moulted progressively later as juveniles, but adults age 4 and older had similar moult start dates that depended on the breeding state of the female. In contrast, moult haulout duration was not constant with age among adults, but instead became shorter with increasing age. Moulting is energetically expensive and differences in the moult haulout duration are possibly due to individual variation in body mass and associated metabolizable energy reserves, although other drivers (e.g. hormones) may also be present. Individual-based data on moult arrival dates and haulout duration can be used as auxiliary data in demographic modelling and may be useful proxies of other important biological parameters such as body condition and breeding history.

## Introduction

Climate change and other global environmental change drivers will continue to alter ecosystem structure, function and resilience this century^1^. Plant and animal species can respond to this environmental change through evolutionary adaptation, dispersal and plasticity in their physiology and behaviour. Phenological shifts are among the most common biological responses to environmental change^2^ with a wide variety of species in different habitats experiencing a shift in the timing of their life-history events during recent decades^3,4^. Phenology, and phenological responses to a changing environment, is of fundamental importance to ecosystem structure and function as it determines how individuals and populations interact with communities and the physical environment^5^. Phenological changes may, for example, drive population dynamics and the evolution of life-histories through its influence on animal movement and reproductive cycles^6^.

Although compelling evidence exists for phenological responses to environmental change, taxonomic, life-history and geographic gaps remain. The phenological responses of mammals to environmental change are poorly studied compared to that of many other taxa. For example, mammals comprised less than 1% of long-term phenological data sets in recent review studies^3,4^. Phenological data sets of vertebrates usually document responses related to the timing of breeding and migration, and rarely the timing of other life-history events^3^. Phenological shifts in one life-history event can, however, influence subsequent life cycle events (e.g. shifts in migration phenology may extend the duration of the breeding season, or shifts in breeding phenology may have carry-over effects for the post-breeding moult)^7^. Most studies of phenological responses are also from terrestrial species, with a bias to northern hemisphere temperate regions. This geographic, life-history and taxonomic bias limits our understanding of the wider ecological and evolutionary consequences of phenological responses to climate change^8^.

Marine mammals respond to environmental change through plasticity of life-history events such as migration, breeding and moulting^9^. For example, shifts in migration phenology of beluga whales (*Delphinapterus leucas*) and moult phenology of Weddell seals (*Leptonychotes weddellii*) are related to seasonal differences in sea ice cover, and consequent variation in availability of prey resources^10,11^. Variation in the availability of prey resources may not only influence the timing of life-history events such as the moult, but also its duration, especially in seals (family Phocidae) that moult on land or ice for several weeks without feeding. For these seals, foraging success and body condition (the amount of blubber energy stored) are thus likely to contribute to variation in the timing and duration of life-history events such as the moult^11,12^.

Southern elephant seals (*Mirounga leonina*; hereafter elephant seals) are circumpolar Southern Ocean marine mammals that breed and moult on land in the austral summer. All elephant seals moult once per year when they shed hair and sheets of cornified epidermis^13^. The moulting of epidermal skin and hair among elephant seals (*Mirounga* spp.) and monk seals (*Monachus* spp.) is unlike that of any other mammal^13,14^. In these seals, and also penguins, the moult is sometimes referred to as a ‘catastrophic moult’ because they replace all their hair (feathers in penguins) while fasting on land or ice^15^. In elephant seals, this process takes about a month and is energetically expensive; adult females tend to lose all the energy gained between breeding and moulting, which amounts to about 150 kg body mass^16,17^. Apart from allocating energy to the growth of new skin and hair, energetic costs are also associated with increased perfusion of blood to peripheral tissues to elevate skin temperature and promote skin and hair growth^16,18^.

In elephant seals, the timing of the moult (starting date) varies by age and according to breeding state in the preceding breeding season^19,20^. Seals moult later as they age, and breeding delays the start of the moult haulout (as in Weddell seals^11^). The duration of the moult haulout is shorter in breeding females than in non-breeding females^12,21^, reflecting the limited ability of breeding females to rebuild energetic reserves during the relatively short foraging phase between breeding and moulting. While these patterns are clear, the environmental factors that influence moult haulout phenology and moult haulout duration have not been studied. Furthermore, it is not known whether changes in the Southern Ocean physical environment^22,23^ may correlate with long-term phenological shifts in the moult.

We aimed to investigate the timing (phenology) and duration of the moult haulout of female elephant seals at Marion Island in the southern Indian Ocean over 32 years (1986–2018). The key questions we address are (1) how do moult phenology and haulout duration vary across age and reproductive categories of female elephant seals; (2) is there evidence for temporal variation (e.g. long-term trends) in the moulting phenology and haulout duration through the study period; and (3) do broad-scale environmental conditions correlate with the timing or duration of the moult haulout?

## Methods

### Southern elephant seal life cycle

Southern elephant seals are wide-ranging marine predators that haul out on land to breed and moult. Every September to October, pregnant females (age three and older) haul out to give birth to a single pup. Female seals do not forage during the breeding season; instead, they metabolize blubber reserves during the lactation period of approximately 3 weeks. At the end of the breeding season, adult females return to sea and spend approximately 70 days foraging and recuperating energy reserves before returning to land (from December to February) to moult^24,25^. All seals moult every year and need to haul out on land and fast during this time. Thus, breeding females enter a second extended fast in the moult. After moulting, adult female elephant seals return to sea to forage for approximately 8 months until the next breeding season^26^. Non-breeding females (juveniles and adult females skipping reproduction^27^) do not haul out during the breeding season, but also moult every year. The juvenile moult somewhat precedes that of adults (November to January)^28^.

### Field methods

Marion Island (46° 54′ S, 37° 45′ E) is part of the Prince Edward Islands group in the southern Indian Ocean. A small population of elephant seals occur here (in 2020, approximately 750 pups were born) that has been part of a capture-recapture study since 1983^29^. During every breeding season, nearly all pups born at the island were uniquely marked at weaning with two hind flipper tags^30^. Since 1984, all beaches where elephant seals frequently haul out were surveyed for tagged seals (Supplementary material S1). Surveys took place weekly during the breeding season and every 10 days during the winter and moult^28^.

During surveys conducted in the moult season, a qualitative moult code was assigned to each tagged elephant seal encountered in the field. Seals were assigned to the pre-moult stage (stage 0) when they were ashore to moult, but, with the old pelage still intact. Trained observers classified actively moulting seals as being in stages 1, 2 or 3, depending on the progression of moult (stage 1: moulted < 1/3rd of the body; stage 2: moulted 1/3rd to 2/3rds of the body; stage 3: moulted > 2/3rds of the body). Fully moulted seals (i.e. those with a new pelage) were assigned to the post-moult stage (stage 4). If an observer was uncertain of the stage of moult, perhaps because the seal was mostly submerged in water or mud, the moult stage for the observation was recorded as ‘unknown’.

This study was conducted under permit from the Director-General: Department of Forestry, Fisheries and the Environment (DFFE), South Africa. Research activities were approved by and carried out in accordance with guidelines provided by the Animal Ethics Committee of the Faculty of Veterinary Science, University of Pretoria, South Africa. All methods are reported in accordance with ARRIVE guidelines.

### Estimating moult haulout timing and duration

We used data of females tagged from 1983 to 2016 and observed from the 1986/87 to 2018/19 moulting seasons (subsequently referred to as 1986–2018). We excluded the first 2 years with moult observations (1984 and 1985) because data on tagged seals were still too limited. Data analyses involved two steps that are detailed below. First, we estimated the moult arrival dates and haulout durations from the observational data. Next, we used the estimated moult arrival dates and moult haulout duration values as response variables in a suite of regression models to investigate which covariates explain variation in moult arrival dates and haulout duration.

The moult arrival dates (expressed as Julian days starting from 1 October) were defined as the earliest date a seal was observed ashore during any moult season. We only used observations of individuals seen in the pre-moult stage to define moult arrival dates (n = 2706 individuals; 5822 observations). Moult haulout durations were defined as the time (in days) that females spent ashore during the moult. In other words, we did not study the duration of “active” or “visible” moult (the time period that a seal was shedding hair and skin) but instead, we estimated the moult haulout duration (the time from arrival ashore to departure to sea). For every individual $i$, we calculated the moult haulout duration as ${duration}_{i}={Last}_{4(i)}-{First}_{0(i)}$, where ${Last}_{4(i)}$ is the last date that the individual was seen in the post-moult stage (stage 4) and ${First}_{0(i)}$ is the first date that the individual was seen in the pre-moult stage (stage 0) (Supplementary material S2). Thus, a moult haulout duration estimate was only obtained if an individual was seen both during the pre-moult stage and the post-moult stage in the same season (n = 2233 individuals; 4612 observations).

A small number of outlying observations occurred in the data. Some of these observations represent atypical moulting events by individual seals that are not representative of the population. The remainder of these observations probably represent errors by field assistants. These observations (< 1% of the data) were excluded from the analyses. For moult arrival dates, we removed observations made before 12 October and arrival dates after 16 February. We excluded from analyses moult haulout durations shorter than 8 days or longer than 56 days.

### Factors affecting the moult

#### Within-year covariates

##### Age

All seals were tagged as pups and thus of known age. We did not consider moulting of the lanugo, which pups shed around weaning. Because we only tagged pups, the monitored population’s age structure changed over the study period. In the 1986 moult season, for example, only females aged 1–3 (those born and tagged in 1983–1985) could be monitored, while in the later years of the study females across the age spectrum (ages ranged from one to 25 years) could be monitored. In our analysis, we represent females’ ages using one of three covariates. The covariate ‘Age (1, 2, …, 15, 16+)’ represents full age variation from age 1 to age 16+ years. Because of small sample sizes in the oldest age classes, females with ages of 16 years and older (n = 65) were grouped in a terminal age class termed ‘16+’. In the covariate ‘Age class (1, 2, 3, 4+)’, 1-, 2- and 3-year-olds were classed separately and females aged 4 years and older were grouped. Females of age 1, 2 and 3 have markedly different body sizes, energetic demands and physiological stressors^31^. One- and two-year-old females are not yet able to breed and only some females reproduce at the age of three, while still undergoing rapid body growth^12^. From the age of four onwards, females have more similar body sizes and food requirements^31^, with many but not all breeding annually^27^. For the third covariate, ‘Age trend (linear)’, we fit a linear regression to test if moult arrival or haulout duration increased (or decreased) linearly with age from age 1 to 16+. Female elephant seals tend to moult later as they age but this has only been studied in females younger than age 10^20^. The influence of age on the moult haulout duration of female elephant seals is largely unknown.

##### Breeding class and breeding state

Breeding delays moult initiation in elephant seals^20^. Because rearing a pup significantly reduces body condition in phocid seals, the later onset of the moult in breeding females may allow them to replenish more energy resources with longer foraging trips before the moult. We used breeding season observations to assign two covariates to females that relate to their breeding history. The covariate ‘breeding class’ made a simple distinction between juveniles (breeding class = 0; females with no history of breeding season presence) and adults (breeding class = 1; assigned to all records after the first breeding season encounter). The covariate ‘breeding state’ distinguished between juveniles (breeding state = 0), breeding adults (breeding state = 1) and non-breeding adults (breeding state = 2). Breeding adults were seen at least once during the breeding season that immediately preceded a specific moult season. Non-breeding adults were adult females (i.e. females with one or more previous breeding records) that were not seen during the breeding season immediately prior to moult. Like in many other synchronously reproducing vertebrates, non-breeding elephant seals are mostly absent from breeding colonies, corresponding to temporary emigration during the breeding season^28^.

The breeding class and breeding state covariates are subject to a low level of misclassification given high breeding season detection probabilities—except for 1998 when breeding observations were poor^28^. To best account for deficient observer effort in 1998, we assumed that all females aged 4 and older were breeding in this year (an assumption that results in the fewest misclassifications of breeding histories in this year).

#### Time-varying covariates

##### Annual trend

Time was modelled as a linear trend to test for a phenological shift in moult arrival dates or a systematic increase or decrease in moult haulout duration over the study period.

##### Environmental covariates

Several environmental covariates were included based on the availability of long-term data, their likely mechanistic links to biological productivity, and their potential importance to the foraging success of female elephant seals. Environmental covariates can be divided into two broad categories: global climatic effects and regional climatic effects. Two global climatic effects, the Southern Annular Mode (SAM) and El Niño Southern Oscillation (ENSO) were used to describe climate variation at the ocean basin scale. Two regional climatic effects, median sea ice extent (SIE) in September and monthly sea surface temperatures (SST) were extracted from within a pre-determined area (latitude 42°–60°S and longitude 10° W–38° E) where female elephant seals from Marion Island frequently forage^32^. We also used mean air temperature (AIR) measured at Marion Island from November to February each year as another explanatory variable for moult haulout duration. Air temperatures may affect moult haulout duration because high peripheral temperature facilitates hair growth^33,34^. Monthly SAM, ENSO and SST values over the period January 1986 to December 2018 were converted into annual means (January to December) for each year of the study. These annual values broadly reflected the environmental conditions that females experienced from one moult to the next and allowed us to detect annual variation and long-term trends in environmental conditions. All environmental covariates were standardized to a mean of 0 and a standard deviation of 1 prior to analysis. Further information about environmental covariates is provided in Supplementary material S3.

### Statistical modelling

We fitted linear mixed-effects models (LMMs) to examine the relationships between the explanatory covariates and moult arrival or haulout duration. To account for repeated individual observations across years and to allow for stochastic annual variation in moult arrival and haulout duration, we fitted LMMs with individual identity and year as random intercepts. The random intercepts partitioned the total variance in moult arrival and haulout duration into between-individual, between-year and residual variance components^35^. The parameters of interest are therefore the population mean and the individual- and temporal variance in moult arrival or haulout duration. Statistical inference was based on model selection (i.e. ranking a set of models using a model selection criterion) and parsimony (i.e. preferring models that represent the data structure with the fewest parameters)^36^.

Models with different combinations of within-year and across-year covariates were analysed to assess the importance of these covariates in explaining variation in the moult arrival dates or moult haulout duration. We were primarily interested in drivers of temporal variations but had to control for between-individual effects (age, age class or age trend; breeding class or breeding state). Therefore, we first considered age and breeding effects to determine which covariates best explained within-year variation. We then used the most parsimonious within-year model as a starting point to which we added across-year covariates (annual trend or the environmental covariates SAM, ENSO, SIE, SST and AIR) that potentially explained inter-annual variation in the moult haulout. Note, however, that because our data were not balanced between years with regards to sample size, age and breeding history, the within-year covariates also explained part of the between-year variance in the data.

#### Model selection

For each model set (moult arrival date and moult haulout duration), plausible models fitted using maximum likelihood (ML) estimation were ranked using Akaike’s Information Criterion (AIC)^37^. The most parsimonious models (with the lowest AIC scores) have the best compromise between variation in the data that are explained by the model and model complexity (number of parameters). Models with △AIC < 2 (the difference between the model with the lowest AIC score and the model in question) indicates similar support from the data^36^. Model parsimony worsens as the △AIC increases and △AIC > 7 indicates strong support for the model with the lower AIC^36^. Mixed models were fitted using the *lme4* package^38^ in R version 3.6.3^39^.

#### Model coefficients and variance components

The most parsimonious models identified in the model selection step were refitted using restricted maximum likelihood (REML) to obtain parameter estimates for moult arrival dates and haulout duration. REML estimation provides unbiased and more reliable variance components than ML in linear mixed-effects models^40^. We used parametric bootstrapping to quantify uncertainty (expressed as 95% confidence intervals) around model estimates^41^. Given that time-varying factors such as environmental covariates cannot explain the within-year variability of moult arrival date and haulout duration, we expected that their contribution to model fit would be relatively low. However, these covariates may still explain important between-year variation and long-term phenology shifts. To quantify the amount of between-individual and -year variance that can be attributed to the covariates in question, we compared the variances of the random individual and random year intercepts between the null model and the models where within-year covariates and across-year covariates were added^35^. If, for example, the covariates explained some of the between-individual and -year variation in moult arrival date it would reduce the variance of these random intercepts. The proportion of the between-individual and -year variance explained by a specific covariate was calculated as the proportional change in variance (PCV), where $PCV\left(\%\right)=1-\left(\frac{{\sigma }_{covariate}^{2}}{{\sigma }_{null}^{2}}\right)\times 100$^41^. Covariates that account for more than 20% of the variation in moult arrival date and haulout duration (PCV ≥ 20%) were considered influential^42^. We summarised the total amount of variance explained using marginal ${R}^{2}$ values (${\text{R}}_{{{\text{LMM}}\left( {\text{M}} \right)}}^{2}$; estimated variance explained by fixed factors) and conditional ${R}^{2}$ values (${\text{R}}_{{{\text{LMM}}\left( {\text{C}} \right)}}^{2}$; estimated variance explained by fixed and random factors)^41^.

## Results

### Moult arrival date

The model with the lowest AIC among those containing only within-year covariates included the additive effect of age class (1, 2, 3 and 4+) and breeding state (juvenile, breeding adult and non-breeding adult) on the moult arrival date (model 10; Table 1). None of the other models containing only within-year covariates were competitive; age class and breeding state were thus retained to investigate the influence of across-year covariates on moult arrival date. Weak evidence of a linear trend (slope = 0.70, 95% CI: − 0.04 to 1.46) towards a later moult arrival date was found (model 12; Table 1). The model including year as a linear trend was most parsimonious and approximately two times better supported by the data compared to the model that excluded any temporal trend (△AIC = 1.45; ѡ = 0.43/0.21 = 2.05). The environmental covariates SAM, ENSO, SIE, and SST did not have any detectable influence on the moult arrival date (Table 1).

**Table 1:** Model selection of the variation in the moult arrival dates of female southern elephant seals over 32 years (1986–2018) at Marion Island.

Model	Fixed effects	np	Deviance	△AIC	ѡ
Null	*I*	4	50,366.24	5354.19	0.00
**Within-year covariates**					
Age effects					
1	Age (1,2,…,15,16 +)	19	45,964.14	982.08	0.00
2	Age class (1,2,3,4 +)	7	45,969.28	963.22	0.00
3	Age trend (linear)	5	48,497.00	3486.94	0.00
Reproductive effects					
4	Breeding state (Juvenile, Breeding adult, Non-breeding adult)	6	46,097.02	1088.96	0.00
5	Breeding class (Juvenile, Adult)	5	46,443.40	1433.34	0.00
Age and reproductive effects					
6	Age + breeding class	20	45,432.50	452.45	0.00
7	Age class + breeding class	8	45,444.40	440.35	0.00
8	Age trend + breeding class	6	46,380.87	1372.81	0.00
9	Age + breeding state	21	44,997.88	19.82	0.00
10	Age class + breeding state	9	45,003.51	1.45	0.21
11	Age trend + breeding state	7	46,000.79	994.73	0.00
**Time-varying covariates**					
12	**Age class** + **breeding state + annual trend**	**10**	**45,000.06**	**0.00**	**0.43**
13	Age class + breeding state + SAM	10	45,003.10	3.04	0.09
14	Age class + breeding state + ENSO	10	45,003.31	3.25	0.08
15	Age class + breeding state + SIE	10	45,003.18	3.12	0.09
16	Age class + breeding state + SST	10	45,002.99	2.94	0.10

Year and individual identity were included as random effects in all models. The most parsimonious model is in bold.

np is the number of estimated parameters, △AIC is the difference between a current model and the model with the lowest AIC value and the Akaike weight (ѡ) represents the relative support for a model given the data and the other models in the selection set. The environmental covariates are Southern Annular Mode (SAM), El Niño Southern Oscillation (ENSO), Sea Ice Extent (SIE) and Sea Surface Temperature (SST).

**Figure 1:**
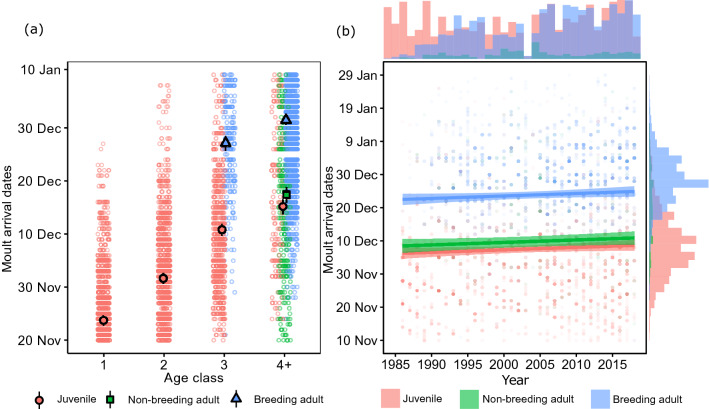
Mean moult arrival dates (and 95% confidence intervals) for female southern elephant seals according to (**a**) age class and breeding state and (**b**) breeding state and time. Estimates were obtained from model 12 (Table 1). Individual data points are plotted on both figures, with the marginal histograms giving the distribution of observations per breeding state.

Female elephant seals moulted later with age, and when they had a pup in the preceding breeding season. The standard deviation of the year random intercept was relatively small compared to the magnitude of the fixed effects when age and breeding effects were included in the model (model 10) (Supplementary material S4). One-year-old females were the first to moult, with a mean arrival date of 23 November (95% CI: 22–24 Nov). Two-year-olds initiated their moult approximately 8 days later, on 1 December (29 Nov–1 Dec), whereas non-breeding females aged 4 years and older moulted nearly a month after 1-year-olds, on 16 December (13–19 Dec). Adult females that reproduced during the year started their moult on average 14 days later than non-breeding females of the same age. Breeding females aged four and older were thus the last group to arrive at the island to moult, on approximately 30 December (29 Dec–1 Jan) (Fig. 1a).

**Figure 2:**
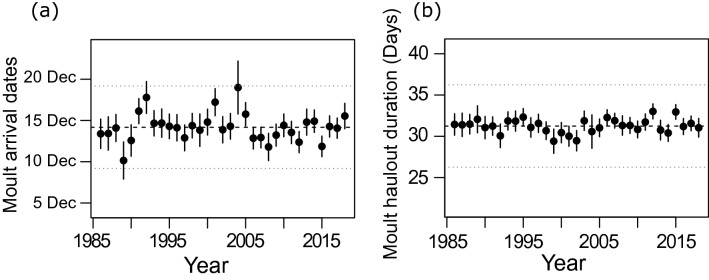
Conditional means (with 95% confidence intervals) of the random effect intercepts that describe the predicted annual deviation in (**a**) moult arrival dates and (**b**) moult haulout duration from the long-term (1986–2018) population-mean value (dashed line). Dotted lines indicates a 5-day shift from the population mean.

The median observed moult arrival date in the first 5 years of the study (1986–1990) was 28 November, while it was 18 December during the last 5 years of the study (2014–2018) (overall median = 14 December, Fig. 2a). This pattern emerged because there was comparatively more tagged juveniles (which haul out earlier to moult) than adults early on in the study population. In contrast, the later study years were characterized by an increased sample of older seals. The delay in moult arrival date was almost entirely accounted for by modelling the change in age structure of the monitored population over time (Fig. 1b). Together, age class and breeding state explained 90.66% of the between-year variance in moult arrival date, and 64.86% of the between-individual variance (Table 2). The percentage among-year variance explained by the model increased only slightly, to 91.49%, with the addition of the linear trend covariate (Table 2). The proportion of the total variance explained by the random and fixed effects was high (${\text{R}}_{{{\text{LMM}}\left( {\text{C}} \right)}}^{2} = 73\%$).

**Table 2:** Variance partitioning of selected linear mixed-effects models of the variation in moult arrival dates of female southern elephant seals at Marion Island (1986–2018).

Model	Null	Model 10 (age class + breeding state)	Model 12 (+ annual trend)
Random effect variances			
Individual	102.84	36.14	36.11
Year	44.88	4.19	3.82
Residuals	250.82	103.80	103.82
Fixed effect variance		234.15	237.50
PCV (Individual)		64.86%	64.89%
PCV (Year)		90.66%	91.49%
PCV (Residuals)		58.62%	58.61%
${\text{R}}_{{{\text{LMM}}\left( {\text{M}} \right)}}^{2}$		0.62	0.62
${\text{R}}_{{{\text{LMM}}\left( {\text{C}} \right)}}^{2}$		0.73	0.73
Deviance	50,366.24	45,003.51	45,000.06
AIC	50,374.24	45,021.51	45,020.06

The random effect variance shows the partitioning of variance among random effects as well as the residual variance. The fixed effect variance shows the amount of variance that has been transferred from the random components to the fixed effects. The PCV (proportion change in variance) indicates how the inclusion of fixed effects has reduced (or when PCV is negative, increased) the variance of each random effect. For the individual random effect, PCV is the percentage of between-individual variance in moult arrival dates explained by covariates. For the year random effect, PCV is the percentage between-year variance in moult arrival dates explained by covariates. The ${\text{R}}_{{{\text{LMM}}\left( {\text{M}} \right)}}^{2}$ value quantifies the amount of variance explained by fixed factors and ${\text{R}}_{{{\text{LMM}}\left( {\text{C}} \right)}}^{2}$ value quantifies the amount of variation explained by both fixed and random factors.

### Moult haulout duration

**Figure 3:**
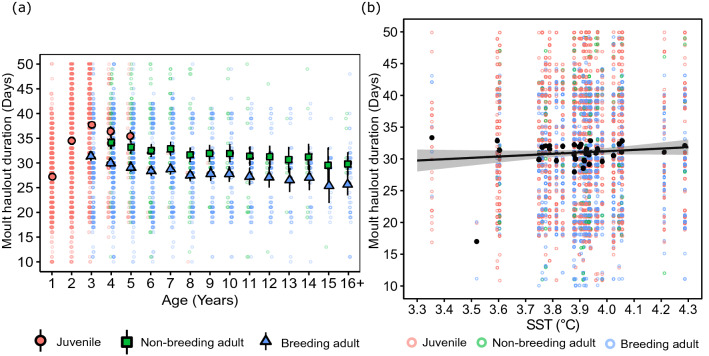
(**a**) Mean moult haulout duration (and 95% confidence intervals) of female southern elephant seals at Marion Island according to age and breeding state. Estimates were obtained from model 9 (Table 3). (**b**) Observed means (black circles) and predicted change (linear regression) in moult haulout duration of female elephant seals with variation in sea surface temperature (SST). The linear regression (with 95% confidence intervals) were obtained from model 16 (Table 3). Individual data points are plotted on both figures (pink—juveniles, green—non-breeding adults and blue—breeding adults).

Moult haulout duration varied with age (Age 1, 2, …, 15, 16+) and breeding state (juvenile, breeding adult and non-breeding adult) in the most parsimonious model that contained only within-year covariates (model 9, Table 3). None of the other models without across-year covariates were competitive. Thus, whereas moult arrival date could be adequately described by a model that distinguished between four age classes (Age 1, 2, 3, 4+), moult haulout duration varied by distinct ages up to age 16+ (Fig. 3a). The model including SST as a linear trend (slope = 0.32, 95% CI: − 0.10 to 0.73; Fig. 3b) had the lowest AIC overall, but the decrease in AIC was unimportant compared to the model containing only age and breeding state (△AIC = 0.25) (model 16, Table 3). None of the other across-year covariates (annual trend, SAM, ENSO, SIE and AIR) explained a meaningful quantity of between-year variation in moult haulout duration (Table 3). Parsimony thus favours predictions of moult haulout duration based on model 9, which contained age and breeding state as fixed effects.

**Table 3:** Model selection of the variation in the moult haulout duration of female southern elephant seals over 32-years (1986–2018) at Marion Island.

Model	Fixed effects	np	Deviance	△AIC	ѡ
Null	*I*	4	33,170.4	813.64	0.00
**Within-year covariates**					
Age effects					
1	Age (1, 2, …, 15, 16+)	19	32,530.52	203.76	0.00
2	Age class (1, 2, 3, 4+)	7	32,585.63	234.87	0.00
3	Age trend (linear)	5	33,082.08	727.31	0.00
Reproductive effects					
4	Breeding state (Juvenile, breeding adult, non-breeding adult)	6	32,950.25	597.48	0.00
5	Breeding class (Juvenile, Adult)	5	32,989.14	634.37	0.00
Age and reproductive effects					
6	Age + breeding class	20	32,381.53	56.77	0.00
7	Age class + breeding class	8	32,415.37	66.60	0.00
8	Age trend + breeding class	6	32,989.07	636.31	0.00
9	Age + breeding state	21	32,323.02	0.25	0.20
10	Age class + breeding state	9	32,363.03	16.27	0.00
11	Age trend + breeding state	7	32,949.65	598.88	0.00
**Time-varying covariates**					
12	Age + breeding state + annual trend	22	32,321.96	1.19	0.13
13	Age + breeding state + SAM	22	32,322.38	1.61	0.10
14	Age + breeding state + ENSO	22	32,321.64	0.88	0.15
15	Age + breeding state + SIE	22	32,322.47	1.70	0.10
16	**Age + breeding state + SST**	**22**	**32,320.77**	**0**	**0.23**
17	Age + breeding state + AIR	22	32,322.57	1.80	0.09

Year and individual identity were included as random effects in all models. The most parsimonious model is in bold.

Model terms are as in Table 1. The environmental covariate AIR is air temperature at Marion Island.

The overall mean moult haulout duration of female elephant seals was 31.25 days (Fig. 2b). Moult haulout duration initially increased with age but decreased gradually from the age of first breeding to age 16+ in both breeding and non-breeding adults (which varied in parallel, as we did not model interactions between age and breeding state). One-year-old females had the shortest mean moult haulout duration of 27.21 (26.52–27.91) days while non-breeding 3-year-old females spent the longest time ashore (37.67 (36.91–38.44) days). The average moult haulout duration of breeding females was 4.15 (3.72–4.58) days shorter than that of non-breeding females of the same age. The mean moult haulout duration of breeding females declined from 31.31 days at age 3 to 25.65 days at age 16+, while non-breeding adult females remained ashore for 34.12 days at age 4 but only 29.81 days at age 16+ (Fig. 3a).

The between-individual variance in moult haulout duration was estimated to be more than three times the between-year variance (Table 4). However, both the between-individual and between-year variability in moult haulout duration was relatively small compared to the influence of age and breeding state on moult haulout duration (the standard deviations of the year (1.09) and individual (1.98) random effects were smaller than all the fixed effect parameter estimates) (Supplementary material S4). The negative PCV values in Table 4 indicate that the between-individual and among-year variances became more pronounced when age, breeding state and SST were accounted for in the model. The SST covariate reduced the variance of the year random effect by 5.57% (model 16 vs. model 9; Table 4) but did not improve the overall fit of the model according to marginal (17%) and conditional (23%) estimators of the explained variance (${{R}^{2}}_{LMM}$).

**Table 4:** Variance partitioning of selected linear mixed-effects models of variation in moult haulout duration of female southern elephant seals at Marion Island (1986–2018).

Model	Null	Model 9 (age + breeding state)	Model 16 (+ SST)
Random effect variances			
Individual	3.48	3.93	3.91
Year	0.95	1.19	1.12
Residuals	73.97	60.75	60.76
Fixed effect variance		13.40	13.35
PCV (Individual)		− 12.79%	− 12.26%
PCV (Year)		− 25.65%	− 18.66%
PCV (Residuals)		17.88%	17.86%
${\text{R}}_{{{\text{LMM}}\left( {\text{M}} \right)}}^{2}$		0.17	0.17
${\text{R}}_{{{\text{LMM}}\left( {\text{C}} \right)}}^{2}$		0.23	0.23
Deviance	33,170.41	32,323.02	32,320.77
AIC	33,178.41	32,365.02	32,364.77

Model terms are as in Table 2.

## Discussion

We found no clear evidence for a shift in moult phenology or haulout duration in elephant seals ashore at Marion Island over a period of 32 years. The annual variation in moult arrival date and haulout duration from 1986 to 2018 was small relative to age and breeding effects, which explained more than 90% of the variance in moult arrival date and 25% in moult haulout duration. Although model selection suggested a correlation between moult haulout duration and sea surface temperature, all environmental covariates we tested, including sea surface temperature, explained minimal variation in the data. Environmental variability affects the distribution and abundance of prey, habitat selection, foraging success and mass gain in marine predators^43^. For example, foraging performance in elephant seals and other upper-trophic level predators such as king penguins (*Aptenodytes patagonicus*) have been linked to variability in large-scale climate indices such as SAM and ENSO^32,44,45^. The environmental conditions that elephant seals experience while foraging (either during the pre-breeding migration or between the breeding- and moulting period) may therefore influence their foraging success and body condition, which in turn may affect moult phenology or haulout duration^18^. Relationships between climatic conditions and phenological variation of life-cycle events exist for several other Southern Ocean marine predators^46,47^. But, our results show that the timing and duration of the moult haulout of elephant seals at Marion Island may be insensitive to changes in environmental conditions in the surrounding Southern Ocean as measured through our covariates.

### Moult arrival dates

We found weak evidence of a linear trend towards a later moult arrival date during the study period, which can probably be explained by residual age and breeding state effects, rather than environmentally driven shifts in moult phenology. No systematic change in moult phenology and the absence of clear relationships between moult arrival date and environmental factors suggest that elephant seal females did not adapt or change the timing of their moult arrival dates under the varying environmental conditions we measured. Phenological insensitivity of breeding seabirds to changes in sea surface temperature^2^ suggests limited environmentally-mediated phenological plasticity in many other marine predators. It is possible that moulting in elephant seals is primarily triggered by other cues, such as photoperiod, that do not vary annually and therefore limits the flexibility in moult phenology per age and breeding state^13^. For example, photoperiod is the main driver of the moult phenology in terrestrial mammals that time their pelage cycles to meet the seasonal environmental demands, although neuroendocrine, endocrine, environmental and intrinsic factors can also play some role to regulate seasonal coat colour moulting^48^.

Our results support earlier work showing that elephant seals moult progressively later as juveniles, but that adults age 4 and older have similar moult start dates that depend on the breeding state of the female^20^. It is worth mentioning here that the moult haulout duration did not follow this pattern; moult haulout duration was not constant with age among adults, but instead became shorter with increasing age. Several studies^33,49,50^ found similar age-related differences in moult arrival dates for another phocid, the Harbour seal (*Phoca vitulina vitulina*). The moult for elephant seals becomes progressively later as juveniles become older and larger, concomitant with the onset and increase in reproductive hormones^13,49^. Breeding females need to rebuild lost energy reserves after participating in the breeding haulout that occurred just 2 months before^25,51^, and thus moult later than juveniles and non-breeders. Breeding history-related differences in the moult timing also occur in Weddell seals, where parturient females initiate moult substantially later than non-parturient females^11^.

### Moult haulout duration

This is the first comprehensive study of moult haulout duration in female elephant seals, with previous studies of moult haulout duration^17^ (from arrival ashore to departure) or the duration of the “active” or “visible” moult^16,52,53^ (i.e. the period during which old hair and skin was shed) restricted to a small number (< 25) of focal individuals (Supplementary material S5). On average, female elephant seals spent 31.25 days ashore during the moult; a haulout duration comparable to that previously obtained from Marion Island (30.4 days; n = 23)^17^ and a small sample of adult female northern elephant seals (*Mirounga angustirostris*)^54^ (32.0 days; n = 8).

Body mass plays an important role in the life histories of female elephant seals^55^. The moult of elephant seals is energetically demanding, with adult females losing about 4.5–5 kg body mass per day^16,52,53^. This amounts to approximately half as much energy expended in the moult than it takes to raise a pup to weaning^16^. Elephant seal yearlings were ashore for the shortest duration during the moult. Because their body size is relatively small, yearlings have less blubber reserves to sustain them during haulouts compared to older seals. Juvenile (non-parturient) 3-year-old females, which approach adult body size^25^, moulted for the longest (37.67 days on average). Breeding females aged 3 years and older had shorter moult haulout durations, perhaps because their breeding effort limited their capacity to accrue energy reserves prior to the moult. Breeding adults spend only approximately 68 days foraging at sea between breeding and moulting^17^. In contrast, juvenile and non-breeding females do not generally haul out during the breeding season and forage continuously. Thus, when arriving ashore to moult, juvenile and non-breeding females will have higher arrival masses than breeders of the same age^12^. However, the body mass of seals may not differ when they depart on their post-moult foraging trips, even if juveniles and non-breeders arrive at higher body mass^12^. Body mass and metabolizable energy differences might thus be the mechanism that explains why different age and breeding classes remain ashore for different durations, even if their “active” moult (the period of visible hair and skin loss, which we did not quantify) do not necessarily differ.

Interestingly, the moult haulout duration of juvenile elephant seals was longer than for non-breeding adults, even though older non-breeders are larger (in body length) and neither were fasting during the breeding season that preceded the moult. In addition, moult haulout duration decreased with age among breeding and non-breeding adults. The shorter moult in non-breeders relative to juveniles, and older females relative to younger females, might suggest that they have a lower body mass at arrival and are thus in poorer body condition. Senescent declines in body mass occur in Weddell seals^56^ and a similar case in female elephant seals might explain why the oldest seals moult the shortest. That said, we do not have data on body mass to support this hypothesis, and we also do not expect senescence to occur in females younger than 16, even though a decline in body mass might precede reductions in survival or breeding^57,58^. Data on body mass trajectories of female elephant seals are thus needed to investigate these ideas as we cannot discount other potential causes of variation in moult haulout duration. Hormones, for example, may be potential drivers of variation in moult haulout duration. The cellular requirements for energy are regulated in part by thyroid hormone and adrenal steroid hormone^59^ that are thought to be implicated in the initiation and control of the annual pelage cycle of pinnipeds^60^. These hormones are important for phocid seal moulting^61^ although gonadal hormones are also thought to be implicated in the annual pelage cycle^60^. Breeding females 3 years and older have established hormonal changes associated with the timing and duration of the moult in relation to the timing of reproductive activities (e.g. suckling of pups). However, in elephant seals there may not be a strong, if at all, relationship between the timing of the moult and the timing of the (delayed) implantation of the blastocyst^13^, and therefore the onset of an active (placental) pregnancy^62^ with its attendant energetic requirements and hormonal changes to maintain and sustain a growing foetus. In addition, breeding adult females moulted later than juveniles of the same age, while juvenile elephant seals also moulted progressively later with increasing age. Therefore, given the wide haulout duration of the moult across all age and breeding classes, it is possible that nutritional status arising from metabolic demands brought about by fasting and reproductive activities during the breeding season, as well as protein requirements for replacing the pelage, is an important factor in determining when the moult haulout takes place^13^.

### Utility of individual-based moult haulout data

We were able to investigate the relationship between the timing of the moult haulout and its duration at an individual level, including how these life-history events correlated with breeding status. Our results show that moult haulout phenology was strongly impacted by age and the reproductive history of individuals. Additionally, our results highlighted between-individual differences in moult haulout duration that were not explained by age and breeding variables but which may potentially occur because individuals differ in how they acquire resources^63^.

We included year random intercepts in addition to individual random effects in our mixed model analysis, allowing variance partitioning between individual and temporal effects. The advantage of this approach is that individual and environmental sources of variances could be decomposed into between-individual and among-year components, to investigate the importance of various fixed effects on moult arrival date and haulout duration. However, many studies of phenology are conducted at the population-level, where all individuals are assumed to behave the same. Population-level studies have several limitations compared to individual-based studies^64^. In population-level studies of phenological change where individual life-history information is not known, the risk is that observed changes in phenology can be result of a change in population structure (e.g. age structure), rather than individual phenological responses to environmental variation^65^. Population-level studies that do not account for selective appearance or disappearance of different categories of individuals over time, perhaps due to environmentally suppressed breeding^66^ or increased anthropogenic adult mortality^67^, may thus reach misleading conclusions about phenological change. Individual-based studies such as ours that can account for age structure and linkage between life-history stages thus enjoy clear advantages over population-level data^64^.

Female elephant seals that reproduced during the breeding season immediately prior to moult had later moult arrival dates and shorter moult haulout durations than those that were not breeding. Such information can be used to assist breeding state assignment in capture-recapture studies with imperfect detection of breeders. Field observations of free-living animals are often associated with imperfect detection (e.g. failure to observe all breeding seals at an island). For example, non-breeding female elephant seals mostly do not attend breeding colonies, and therefore missing observations can represent either a detection error or a skipped breeding year. In such cases, multiple data sources (e.g. observations made at other times of the year) have the potential to produce inferences that are less biased and more precise^28^. At Marion Island, high observation effort in the breeding season results in few failures of detection and high confidence in breeding state assignment^28^. Our results show that the correlation between breeding status, moult arrival date and moult haulout duration can be used to revise breeding states of individuals where more uncertainty exists in breeding states. This approach (moult arrival dates) was used to assign Macquarie Island female elephant seals with uncertain breeding histories to either the breeder or non-breeder states^68^. A similar approach was used to correct capture-recapture breeding estimates of grey seals, *Halichoerus grypus,* using body mass measurements^63^. While body mass measurements at the start of the moult may also be indicative of elephant seal breeding activity^12^, those based on the timing and duration of moult haulout are easier to obtain for a large number of individuals.

Our results provide empirical data about the moult haulout duration per age and breeding state for use in life-history theory models^69^. Furthermore, given that it is far easier to monitor annual changes in moult haulout duration than changes in body mass, future studies should evaluate whether moult haulout duration can be used as a proxy of body mass (condition) of female elephant seals at the start of the moult. Previous studies have indicated that females that bred prior to the moult have lower body mass when they returned to land to moult as compared to same-aged non-breeders^12^. If the shorter moult haulout duration of breeding females is driven by energetic constraints, moult haulout duration may be used to track individual or temporal changes in the energetic state of females when they haul out to moult. Accounting for age and breeding state will be crucial, but the residual variation in moult haulout duration may represent temporal changes in body condition, foraging success or prey availability.

Phenotypic flexibility in each life-history event is constrained by life histories^15^. For instance, long-distance migrators including elephant seals and many bird species may alter their life history phenology less than resident species because they cannot access cues on their feeding or breeding grounds. Additionally, the additive durations of the moult, breeding, and foraging periods may not allow for one event to be longer. In general, the phenological triggers of these life-history events as well as phenological links between life history events are poorly studied, especially in marine species^70,71^. We show that southern elephant seal moult is strongly impacted by age and breeding, but less impacted by climate and oceanographic metrics. Another potentially fruitful avenue of research is comparing and contrasting the phenological flexibility in moult versus breeding. In northern elephant seals, for example, parturition takes place on average 5 days after arrival on the beach but the start of the fur replacement process likely occurs much later after arrival on the beach^72^. Likewise, northern elephant seal females tend to depart immediately after weaning their pups, whereas they wait up to several weeks after finishing the moult to depart^72^. Therefore, the moult haulout may confer more flexibility than the breeding haulout; however, to our knowledge, individual variability in moult versus breeding timing has not been directly compared. Breeding and moult phenology events should thus also be studied in combination to investigate how fitness of long-lived species is affected by food availability, environmental changes and linkages between life cycle events.

## Supplementary Information

Supplementary Information.

## Data Availability

The data that support the findings of this study are available from the corresponding author on reasonable request.
