# Publisher Correction: Determinants of moult haulout phenology and duration in southern elephant seals

**DOI:** 10.1038/s41598-021-93678-8

**Published:** 2021-07-01

**Authors:** Leandri de Kock, W. Chris Oosthuizen, Roxanne S. Beltran, Marthán N. Bester, P. J. Nico de Bruyn

**Affiliations:** 1 Department of Zoology and Entomology, Mammal Research Institute, University of Pretoria, Private Bag X20, Hatfield, 0028 South Africa; 2 Marine Apex Predator Research Unit, Institute for Coastal and Marine Research and Department of Zoology, Nelson Mandela University, Port Elizabeth, 6031 South Africa; 3 Department of Ecology and Evolutionary Biology, University of California Santa Cruz, 115 McAllister Way, Santa Cruz, CA 95060 USA

Correction to: *Scientific Reports* 10.1038/s41598-021-92635-9, published online 25 June 2021

The original version of the Article contained an error in the Author Information section.

“These authors contributed equally: Leandri de Kock and P.J. Nico de Bruyn.”

now reads:

“These authors contributed equally: Leandri de Kock and W. Chris Oosthuizen.”

The original Article has been corrected.

