# Supplementary material for: Determinants of moult haulout phenology and duration in southern elephant seals
Source: Sci Rep. 2021 Jun 25;11:13331. doi: 10.1038/s41598-021-92635-9 (PMC8233432; doi:10.1038/s41598-021-92635-9)
Supplement: Supplementary file 1 — Supplementary Information. [file 41598_2021_92635_MOESM1_ESM.docx]

**Determinants of moult haulout phenology and duration in southern elephant seals**

**Leandri de Kock^1†^, W. Chris Oosthuizen^1,2†*^, Roxanne S. Beltran^3^, Marthán N. Bester^1^, P.J. Nico de Bruyn^1^**

^1^Mammal Research Institute, Department of Zoology and Entomology, University of Pretoria, Private Bag X20, Hatfield 0028, South Africa.

^2^Marine Apex Predator Research Unit, Institute for Coastal and Marine Research and Department of Zoology, Nelson Mandela University, Port Elizabeth 6031, South Africa

^3^Department of Ecology and Evolutionary Biology, University of California Santa Cruz, 115 McAllister Way, Santa Cruz, California, 95060, United States of America

^†^These authors contributed equally to this work

* [wcoosthuizen@zoology.up.ac.za](mailto:wcoosthuizen@zoology.up.ac.za)

Supplementary Material S1 – S5

Supplementary Material S1


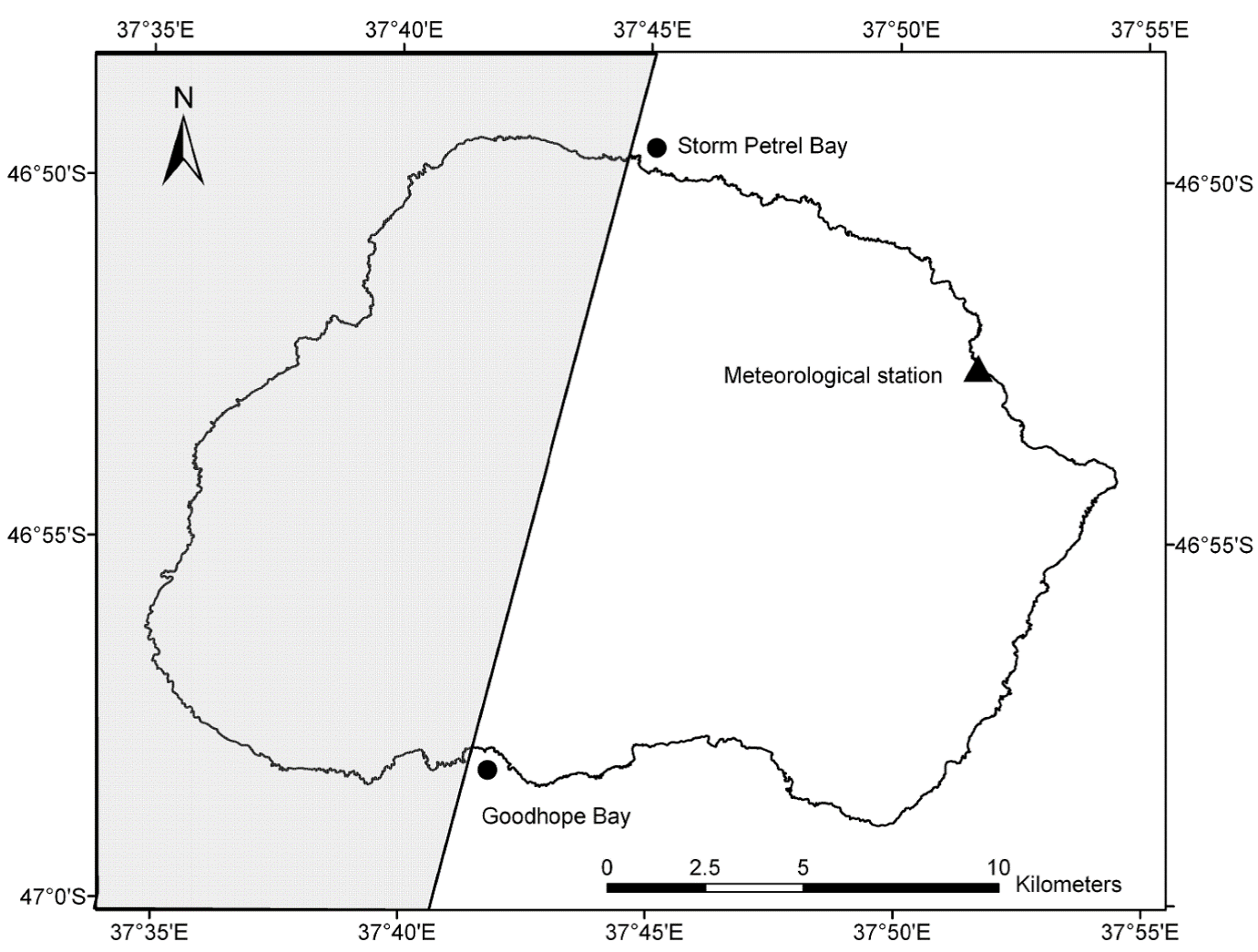


Figure S1.1 Map of Marion Island (290 $\mathrm{km}^{2}$). The main study area for the southern elephant seal capture-recapture programme is on the eastern half of the island, from Storm Petrel Bay in the north to Goodhope Bay in the south. Elephant seals predominantly haul out on relatively small pebble beaches and vegetated areas along the northern and eastern shores. Much of the western and southern coastline of Marion Island is exposed to powerful wave action. Here, the coast mostly consists of cliffs or narrow rocky beaches that are either inaccessible to elephant seals or are simply not preferred by them (Mulaudzi et al., 2008).

**References**

Mulaudzi, T. W., Hofmeyr, G. J. G., Bester, M. N., Kirkman, S. P., Pistorius, P. A., Jonker, F. C., Makhado, A. B., Owen, J. H. & Grimbeek, R. J. 2008. Haulout site selection by southern elephant seals at Marion Island. *African Zoology,* 43**,** 25-33.

Supplementary Material S2


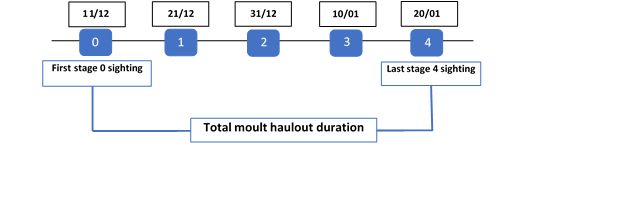


Figure S2.1 Moult scores (numbers in blue boxes) of a hypothetical female southern elephant seal seen during moulting season surveys at Marion Island. Black boxes represent the dates (dd/mm) when specific moulting stages (scores) were observed. The moult arrival date is the first stage zero sighting. The moult haulout duration can be calculated by subtracting the date when the female was first seen in stage 0 from the last date the female was seen in stage 4.

Supplementary Material S3.

Details of the environmental variables used to describe moult arrival date and haulout duration of female elephant seals at Marion Island.


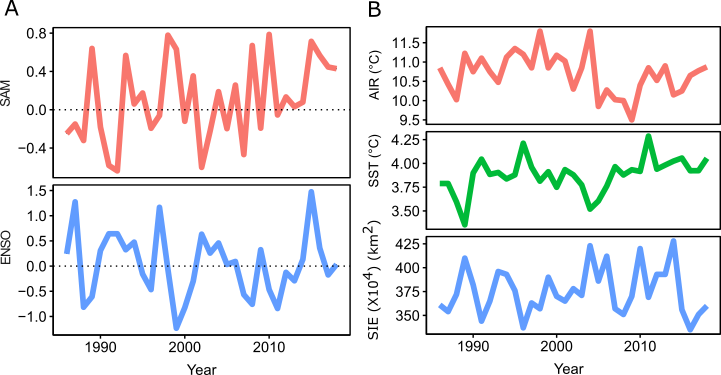


Figure S3.1 Patterns of variation in environmental covariates fitted as explanatory variables in linear mixed-effect models of female elephant seal moult arrival date and moult haulout duration (1986 – 2018). A) Global climatic effects (Southern Annular Mode (SAM) and El Niño Southern Oscillation (ENSO)). B) Regional climatic effects (Air Temperature at Marion Island (AIR), Sea Surface Temperature (SST) and Sea Ice Extent (SIE)).

*Global climatic effects:*

*Southern Annular Mode (SAM) –* SAM provides an index of the oceanographic changes that occur due to fluctuating wind patterns (Lefebvre and Goosse, 2005, McMahon et al., 2015). SAM describes the north/south shifts in westerly winds surrounding Antarctica as its mode changes between positive and negative (Hauck et al., 2013). SAM influences large-scale biological productivity in the ocean, with positive phases of SAM leading to higher ocean productivity south of the Antarctic Polar Front (Hindell et al., 2012). The winds associated with SAM drive the circulation of the Antarctic Circumpolar Current (ACC) and causes upwelling of warm circumpolar deep water, thereby fueling the production of phytoplankton biomass (Hauck et al., 2013, Spence et al., 2014). Monthly SAM index values (January 1986 - December 2018) (<https://www.cpc.ncep.noaa.gov/>) were converted into annual means by averaging the monthly values from January in year $t$ to December in year $t$ to provide a broad-scale index which represents the climatic conditions experienced by females during their foraging migrations.

*El Niño Southern Oscillation (ENSO) -* ENSO events occur in two to seven-year cycles with irregular variations in sea temperatures (Holmgren et al., 2006). ENSO, an important phenomenon in tropical regions, also influence the weather and oceanic conditions in high latitude areas of the Southern hemisphere (Schine et al., 2016). However, ENSO-related environmental changes are variable in different regions of Antarctica (Stammerjohn et al., 2008). ENSO events have substantial impacts on surrounding ecosystems in Antarctica such as affecting the physical environments (e.g. sea ice) and driving the biological environments (e.g. productivity) within which elephant seals forage (Carleton and Carpenter, 1990, Atkinson et al., 2004). For example, the survival of juvenile elephant seals at Macquarie Island was dependent on ENSO (McMahon and Burton, 2005). ENSO-related effects may disrupt nutrient balances and thus productivity which will ultimately affect the primary and secondary production available to marine predators (Sarmiento et al., 2004). The variability of ocean productivity is being linked to the variations in the foraging successes of elephant seals (Bradshaw et al., 2004). As before, ENSO values (January 1986 - December 2018) (<https://origin.cpc.ncep.noaa.gov>) were converted into annual means before analysis.

*Regional climatic effects:*

*Sea ice extent (SIE) –* Sea ice is important for primary and secondary productivity in Antarctic waters (Barbraud and Weimerskirch, 2006). Less sea ice may lead to the decrease in prey availability (e.g. sub-Antarctic krill, *Euphausia* spp.) on which elephant seals feed (van den Hoff et al., 2003, Lübcker et al., 2017). To quantify the effects of sea ice extent on the moulting phenology of elephant seals through possible links with foraging success, we used the median sea ice extent (${km}^{2})$ from the Indian Ocean range. The median sea ice extent for September was used as this is when sea ice in our study region reaches or is close to its annual maximum extent. The monthly sea ice extent data (September 1986 – September 2018) (<https://nsidc.org/data/NSIDC-0192/versions/3>) was derived from the integration of the daily Electrical Scanning Microwave Radiometer (ESMR), Scanning Multichannel Microwave Radiometer (SMMR), Special Sensor Microwave/Imager (SSM/I), and the Special Sensor Microwave Imager/Sounder (SSMIS), Version 3 (Stroeve and Meier, 2018).

*Sea surface temperature (SST) –* SSTs are known to influence foraging areas and foraging success of elephant seals (Bradshaw et al., 2004, Simmons et al., 2007, Cox et al., 2020). Monthly SST values were extracted from the remotely sensed NOAA 1/4° Daily Optimum Interpolation Sea Surface Temperature data set on the NOAA ERDDAP server (<https://www.ncdc.noaa.gov/oisst>). The monthly SST values were extracted in the area (latitude 42°S to 60°S and longitude 10°W to 38°E) where female elephant seals of Marion Island frequently forage (as determined from satellite tracking data of females from Marion Island; Oosthuizen et al. 2015). The monthly SST values (January 1986 – December 2018) were converted into annual SST mean values for each year of the study period.

*Air temperature at Marion Island (AIR) –* Epidermal temperature is an important aspect in the moult of seals (Daniel et al., 2003). To facilitate hair growth, elephant seals maintain high peripheral temperatures through blood flow and avoid vasoconstriction in response to changing weather conditions (Chaise et al., 2018). In Weddell seals, ambient temperatures provided a selective pressure on the moult phenology (Walcott et al., 2020). We included air temperature (°C) measured at Marion Island as a factor potentially affecting the moult duration between years. We used the daily maximum ground-level air temperatures collected at the meteorological station on the northeastern side of Marion Island (November 1986 - February 2019) and made available by the South African Weather Service (SAWS). We only used data for the months that elephant seal females were hauled out to moult (November to February), and calculated a mean temperature for each year. Air temperatures were not thought to influence seals at sea and thus were not considered an appropriate covariate for moult arrival date.

**References**

Atkinson, A., Siegel, V., Pakhomov, E. & Rothery, P. 2004. Long-term decline in krill stock and increase in salps within the Southern Ocean. *Nature,* 432**,** 100-103.

Barbraud, C. & Weimerskirch, H. 2006. Antarctic birds breed later in response to climate change. *Proceedings of the National Academy of Sciences,* 103**,** 6248-6251.

Bradshaw, C. J., Hindell, M. A., Sumner, M. D. & Michael, K. J. 2004. Loyalty pays: potential life-history consequences of fidelity to marine foraging regions by southern elephant seals. *Animal Behaviour,* 68**,** 1349-1360.

Carleton, A. M. & Carpenter, D. A. 1990. Satellite climatology of ‘polar lows’ and broadscale climatic associations for the Southern Hemisphere. *International Journal of Climatology,* 10**,** 219-246.

Chaise, L. L., Prinet, I., Toscani, C., Gallon, S. L., Paterson, W., Mccafferty, D. J., Théry, M., Ancel, A. & Gilbert, C. 2018. Local weather and body condition influence habitat use and movements on land of molting female southern elephant seals (*Mirounga leonina*). *Ecology and Evolution,* 8**,** 6081-6090.

Cox, S. L., Authier, M., Orgeret, F., Weimerskirch, H. & Guinet, C. 2020. High mortality rates in a juvenile free-ranging marine predator and links to dive and forage ability. *Ecology and Evolution,* 10**,** 410-430.

Daniel, R. G., Jemison, L. A., Pendleton, G. W. & Crowley, S. M. 2003. Molting phenology of harbor seals on Tugidak Island, Alaska. *Marine Mammal Science,* 19**,** 128-140.

Hauck, J., Völker, C., Wang, T., Hoppema, M., Losch, M. & Wolf‐Gladrow, D. A. 2013. Seasonally different carbon flux changes in the Southern Ocean in response to the southern annular mode. *Global Biogeochemical Cycles,* 27**,** 1236-1245.

Hindell, M. A., Bradshaw, C. J., Brook, B. W., Fordham, D. A., Kerry, K., Hull, C. & McMahon, C. R. 2012. Long‐term breeding phenology shift in royal penguins. *Ecology and Evolution,* 2**,** 1563-1571.

Holmgren, M., Stapp, P., Dickman, C. R., Gracia, C., Graham, S., Gutiérrez, J. R., Hice, C., Jaksic, F., Kelt, D. A. & Letnic, M. 2006. A synthesis of ENSO effects on drylands in Australia, North America and South America. *Advances in Geosciences,* 6**,** 69-72.

Lefebvre, W. & Goosse, H. 2005. Influence of the Southern Annular Mode on the sea ice-ocean system: the role of the thermal and mechanical forcing. *Ocean Science,* 1**,** 145-157.

Lübcker, N., Reisinger, R. R., Oosthuizen, W. C., De Bruyn, P. N., Van Tonder, A., Pistorius, P. A. & Bester, M. N. 2017. Low trophic level diet of juvenile southern elephant seals *Mirounga leonina* from Marion Island: a stable isotope investigation using vibrissal regrowths. *Marine Ecology Progress Series,* 577**,** 237-250.

McMahon, C. R. & Burton, H. R. 2005. Climate change and seal survival: evidence for environmentally mediated changes in elephant seal, *Mirounga leonina*, pup survival. *Proceedings of the Royal Society B: Biological Sciences,* 272**,** 923-928.

McMahon, C. R., New, L., Fairley, E., Hindell, M. & Burton, H. 2015. The effects of body size and climate on post‐weaning survival of elephant seals at Heard Island. *Journal of Zoology,* 297**,** 301-308.

Sarmiento, J. L., Gruber, N., Brzezinski, M. & Dunne, J. 2004. High-latitude controls of thermocline nutrients and low latitude biological productivity. *Nature,* 427**,** 56-60.

Schine, C. M., Van Dijken, G. & Arrigo, K. R. 2016. Spatial analysis of trends in primary production and relationship with large‐scale climate variability in the Ross Sea, Antarctica (1997–2013). *Journal of Geophysical Research: Oceans,* 121**,** 368-386.

Simmons, S. E., Crocker, D. E., Kudela, R. M. & Costa, D. P. 2007. Linking foraging behaviour of the northern elephant seal with oceanography and bathymetry at mesoscales. *Marine Ecology Progress Series,* 346**,** 265-275.

Spence, P., Griffies, S. M., England, M. H., Hogg, A. M., Saenko, O. A. & Jourdain, N. C. 2014. Rapid subsurface warming and circulation changes of Antarctic coastal waters by poleward shifting winds. *Geophysical Research Letters,* 41**,** 4601-4610.

Stammerjohn, S. E., Martinson, D., Smith, R., Yuan, X. & Rind, D. 2008. Trends in Antarctic annual sea ice retreat and advance and their relation to El Niño–Southern Oscillation and Southern Annular Mode variability. *Journal of Geophysical Research: Oceans,* 113.

Stroeve, J. & Meier, W. N. 2018. Sea Ice Trends and Climatologies from SMMR and SSM/I-SSMIS, Version 3. [September 1984 - September 2018]. Boulder Colorado USA. NASA National Snow and Ice Data Center Distributed Active Archive Center. doi: https://doi.org/10.5067/IJ0T7HFHB9Y6 [17/04/2020].

Van Den Hoff, J., Burton, H. & Davies, R. 2003. Diet of male southern elephant seals (*Mirounga leonina* L.) hauled out at Vincennes Bay, East Antarctica. *Polar Biology,* 26**,** 27-31.

Walcott, S. M., Kirkham, A. L. & Burns, J. M. 2020. Thermoregulatory costs in molting Antarctic Weddell seals: impacts of physiological and environmental conditions: Themed Issue Article: Conservation of Southern Hemisphere Mammals in a Changing World. *Conservation physiology,* 8**,** coaa022.

Supplementary material S4

Table S4.1. Moult arrival date fixed-effect estimates (and 95% confidence intervals) obtained from Model 12, Table 1 (main manuscript).


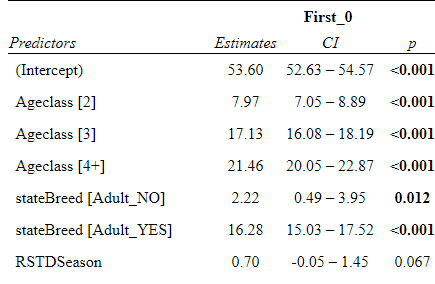


Table S4.2. Moult haulout duration fixed-effect estimates (and 95% confidence intervals) obtained from model 16, Table 3 (main manuscript).


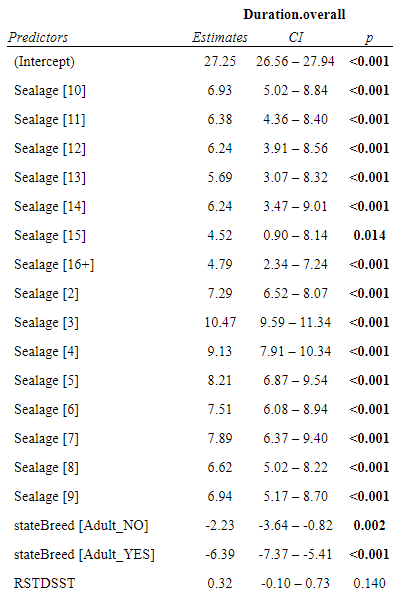


Supplementary material S5

Table 5.1. Comparison of the moult duration, and moult haulout duration, for four southern elephant seal (*Mirounga leonina*) populations. Postma et al. (2013) studied the moult haulout duration (time from arrival ashore to departure to sea), which is similar to our work. By contrast, Hindell et al. (1994) and Carlini et al. (1999) studied the moult duration, i.e. they stopped monitoring when seals had completed the “active” moult (i.e., when the old hair was shed and a new coat grown).. Boyd et al. (1993) states that “it was not possible to measure the duration of the moulting fast in terms of the total time seals spent at South Georgia”. Their duration represents the period between the start of the moult, and physical recaptures of fully moulted seals and we thus interpret it as the moult duration.

| Southern elephant seal population | Moult duration (Mean ± SD) | Moult haulout duration  (Mean ± SD) | Number of females studied (n) | Reference |
| --- | --- | --- | --- | --- |
| Marion Island |  | 31.3 ± 8.9 days | 4612 | (This study) |
| Marion Island |  | 30.4 ± 6.8 days | 23 | (Postma et al., 2013) |
| King George Island | 25.7 ± 4.3 days |  | 9 | (Carlini et al., 1999) |
| South Georgia | 21.0 ± 4.4 days |  | 19 | (Boyd et al., 1993) |
| Macquarie Island | 16.8 ± 3.1 days |  | 13 | (Hindell et al., 1994) |

**References**

Boyd, I., Arnbom, T. & Fedak, M. 1993. Water Flux, Body Composition, and Metabolic Rate during Molt in Female Southern Elephant Seals (*Mirounga leonina*). *Physiological Zoology,* 66**,** 43-60.

Carlini, A., Marquez, M., Daneri, G. & Poljak, S. 1999. Mass changes during their annual cycle in females of southern elephant seals at King George Island. *Polar Biology,* 21**,** 234-239.

Hindell, M. A., Slip, D. J. & Burton, H. R. 1994. Body mass loss of moulting female southern elephant seals, *Mirounga leonina*, at Macquarie Island. *Polar Biology,* 14**,** 275-278.

Postma, M., Bester, M. N. & De Bruyn, P. N. 2013. Spatial variation in female southern elephant seal mass change assessed by an accurate non-invasive photogrammetry method. *Antarctic Science,* 25**,** 731-740.
